# Stamps and Hospitals

**Published:** 1898-10-01

**Authors:** 


### STAMPS AND HOSPITALS.

Sir,—Now that public attention is again directed to the new issue of hospital stamps for 1898, I beg to make a suggestion that may be of use. New South Wales has issued hospital stamps for 1s. and 2s. 6d., the first giving 11d., and the second 2s. 3½d. profit to the hospital. These stamps are beautiful and much sought by the public and collectors, and their value greatly increases. Now we should follow the colonial example with stamps of face value from 1s., 2s. 6d., 5s., and 10s., the postage or carrying power being respectively 1d., 2½d., 5d., and 10d. Then thousands and thousands would be sold above the common demand; people would send them to their friends and also keep them unused, while collectors would be extensive patrons and safe custodians. Without doubt the colonials have issued some beautiful stamps and on the right plan to benefit the hospitals.—I am, sir, your obedient servant, J. W. Palmer.

281, Strand, W.C., September 17th.

⁂ Mr. Palmer is evidently unaware that the British Post Office has so far refused to permit any such scheme as that he suggests. Practically the whole face value of the Prince of Wales's Hospital Stamps is credited to the hospitals, for the expenses of the issue are immaterial. If everybody interested in the hospitals would buy or induce one person to purchase a stamp album and the four stamps the whole issue, which is this year a small and limited one, would be sold out in a fortnight.—Ed. *T. H.*

